# Changes in Lifestyle Habits among Adolescent Girls after FitSpirit Participation

**DOI:** 10.3390/ijerph17124388

**Published:** 2020-06-18

**Authors:** Karine Paiement, Vicky Drapeau, Jo-Anne Gilbert, Jean Lemoyne, Nicolas Moreau, Johana Monthuy-Blanc, Jonathan Tremblay, Valérie Marcil, Marie-Eve Mathieu

**Affiliations:** 1 École de Kinésiologie et des Sciences de L’activité Physique, Université de Montréal, 2100 boul. Édouard-Montpetit, Montréal, QC H3T 1J4, Canada; karine.paiement.1@umontreal.ca (K.P.); j.gilbert@umontreal.ca (J.-A.G.); jonathan.tremblay@umontreal.ca (J.T.); 2 Département de Nutrition, Université de Montréal, 2405 chemin de la Côte-Sainte-Catherine, Montréal, QC H3T 1A8, Canada; valerie.marcil@umontreal.ca; 3 Département D’éducation Physique, Université Laval, 2300 rue de la Terrasse, Québec, QC G1V 0A6, Canada; vicky.drapeau@fse.ulaval.ca; 4 Département des Sciences de L’act. Physique, Université du Québec à Trois-Rivières, 33351 boul. des Forges, Trois-Rivières, QC G8Z 4M3, Canada; jean.lemoyne@uqtr.ca; 5 School of Social Work, University of Ottawa, 120 University Private, Ottawa, ON K1N 6N5, Canada; nicolas.moreau@uottawa.ca; 6 GR2TCA-Loricorps, Groupe de Recherche Transdisciplinaire des Troubles du Comportement Alimentaire, Département des Sciences de l’Éducation, Université du Québec à Trois-Rivières, 33351 boul. des Forges, Trois-Rivières, QC G8Z 4M3, Canada; johana.monthuy-blanc@uqtr.ca; 7 Centre de recherche du CHU Sainte-Justine, 3175 chemin de la Côte-Sainte-Catherine, Montréal, QC H3T 1C5, Canada

**Keywords:** physical activity, school-based intervention, after-school programs, extracurricular programs, adolescence, adolescent girls, female, public health, health behaviors, lifestyle habits

## Abstract

Adolescence is a crucial time in the development and maintenance of lifestyle habits. Interventions to improve health-related behaviors are important, including those that can contribute to an increase in physical activity (PA). During adolescence, PA typically decreases with age, particularly in girls. The FitSpirit program offers services that help Canadian schools from Quebec and Ontario implement PA interventions for adolescent girls. This study aimed to evaluate changes in participants’ PA levels and lifestyle habits (sedentary time, sleep duration and eating habits) and to assess whether these changes depended on adherence to the Canadian 24-Hour Movement Guidelines and Canada’s Food Guide recommendations at enrollment. At the time of FitSpirit registration (between December 2018 and March 2019) and in May/June 2019, 73 participants answered online questionnaires. The participants reported improvements, with an increase in the number of days with PA and a decrease in daily consumption of sweets. The greatest changes were observed in those who did not adhere to the Canadian recommendations before enrollment and who significantly increased their number of days with PA and their consumption of fruits and vegetables, and decreased their screen time. In conclusion, participation in FitSpirit improved several health behaviors among adolescent girls, particularly those who did not comply with the Canadian recommendations at enrollment.

## 1. Introduction

As young children and adolescents grow and develop, the benefits of healthful lifestyle habits are numerous. A healthy 24 hours includes high levels of physical activity (PA), low levels of sedentary behaviors and sufficient sleep each day [1]. Associations between PA and health outcomes including lower adiposity indicators, favorable cardiometabolic biomarkers, better physical fitness, favorable indicators of bone health, greater well-being, greater motor skill development and lower psychological distress have been well-documented [2,3,4,5,6,7]. The literature also supports the relationship between sedentary time including recreational screen time (TV viewing, computer use, cell phone use and handheld video game use) and adverse health outcomes such as obesity, adverse cardiometabolic risk factors, lower physical fitness, behavioral problems, lower self-esteem and poorer academic achievement [7,8,9]. Sleep is an important contributor to physical and mental health. Longer sleep duration is associated with lower adiposity indicators, better emotional regulation, better academic achievement and greater well-being [10]. Increasing evidence indicates that short sleep duration and poor sleep quality are associated with increased food intake, poor diet quality and excess body weight in adolescents [11,12]. The Canadian 24-Hour Movement Guidelines are the first evidence-based guidelines to address the whole day for children and youth. They include recommendations about moderate to vigorous physical activity (MVPA) (at least 60 min/day), light PA (several hours/day), sleep (9–11 h/night for those aged 5–13 and 8–10 h/night for those aged 14–17 years) and sedentary behavior (<2 h/day of recreational screen time) [1].

Among eating habits, fruit and vegetable consumption is an important part of a healthful diet and is associated with lower risks of developing cancer, cardiovascular disease and other chronic diseases in adulthood, as well as with benefits in weight management [13,14,15,16,17,18,19,20]. The recommendations for a healthful diet can be more effective if they are contextualized in dietary guidelines. Canada’s Food Guide is an educational tool developed by Heath Canada to help people follow a healthy diet [21]. It includes plant-based foods (fruits, vegetables, legumes and whole grain foods) and limits foods high in sodium, sugars or saturated fat. The 2007 Canada Food Guide includes recommendations about daily consumption of dietary groups such as vegetables and fruits (6 portions/day for girls aged from 9–13 and 7 portions/day for those aged 14–18 years) [22]. Healthful eating habits include eating vegetables, fruits and dairy; drinking water; and eating breakfast in the morning, whereas eating habits that can be harmful include the consumption of sugar-sweetened beverages, sweets and fast food [23].

Adolescence is a crucial period for the development and maintenance of healthful lifestyle habits, and thus much emphasis has been placed on interventions to improve health-related behaviors at a young age [24,25,26]. This is especially important, given that PA decreases with age, especially in girls, whereas sedentary behaviors increase [26,27,28]. Moreover, insufficient sleep is common among adolescents [29]. In recent decades, the deterioration in eating habits has included a rapid increase in the consumption of highly processed/energy-dense foods and a decrease in healthful eating habits, such as fruit and vegetable consumption [30,31,32,33]. Canadian adolescents report low frequency of fruit and vegetable consumption and a lower frequency of consumption is associated with higher body mass index (BMI) [34]. In adolescent girls from Quebec, in Canada, the proportion of students consuming the recommended number of servings of fruits and vegetables is 26% [23].

School-based interventions targeting overweight and obesity prevention and treatment are conducive to the development of healthful lifestyle habits among young people because they have the potential to reach most children and adolescents [35,36]. Recent systematic reviews have assessed interventions aimed at increasing PA in adolescent girls across schools and community settings [37,38,39]. The authors have reported variable effect sizes and mixed results regarding the effectiveness of the interventions. However, they have also highlighted that interventions are more likely to be effective in increasing PA in adolescents (12–15 years old) if they use a multi-component approach and target both PA and sedentary behaviors. A multi-component approach includes comprehensive programs that facilitate changes in behavior (physical activity, sedentary and dietary behaviors) by using a number of methods (support components, individual components, choice components and educational and environmental components) to target and change unhealthy patterns [37]. Although many programs worldwide have aimed to increase PA in youth, few have focused solely on adolescent girls, and even fewer have been evaluated for their potential effects on a wide range of lifestyle habits [40,41].

FitSpirit is a non-profit organization that has helped Canadian schools implement interventions to promote PA among adolescent girls since 2007 [42]. FitSpirit offers a multicomponent approach and reaches nearly 12,000 girls annually. In fact, FitSpirit aims to raise adolescent girls’ awareness of the benefits of an active lifestyle by increasing motivation and enjoyment of regular PA. Previous research has shown promising results regarding motivational outcomes associated with FitSpirit [43]. However, little is known about participant lifestyle and the changes brought about by FitSpirit. The specific goals of this study were two-fold: (1) to evaluate changes in participants’ PA levels and lifestyle habits (sedentary time, sleep duration and some eating habits) and (2) to assess whether these changes are associated with adherence to the Canadian 24-Hour Movement Guidelines and Canada’s Food Guide recommendations before participant enrollment in the program. We hypothesized that: (1) participation in FitSpirit would promote healthful lifestyles in adolescent girls, including higher PA levels and improvements in sedentary behaviors, sleep duration and eating habits, and (2) the lifestyle changes would be associated with adherence to the Canadian recommendations before enrollment in the program, and the magnitude of changes would be greater for girls with poorer adherence to the recommendations at the beginning of the program.

## 2. Materials and Methods

### 2.1. Study Design and Participants

FitSpirit offers tools and services to support schools in engaging girls in PA. Every year, each school chooses from among a selection of girls-only activities offered by FitSpirit (Table 1). Readers are invited to visit the website www.fitspirit.ca for further information about the program.

In 2018–2019, 285 schools across eastern Canada (177 in Quebec and 108 in Ontario) participated in FitSpirit. After schools registered as FitSpirit partner schools, a member of the FitSpirit team helped them enroll girls from their school in the program. FitSpirit participants 14 years of age and older were eligible to enroll in the study by providing written informed consent. Younger participants were eligible if their parents provided written informed consent. The study was conducted in accordance with the Declaration of Helsinki, and ethics approval was obtained from the Université de Montréal ethical committee (Comité d’éthique de la recherche en santé, #16–160-CERES-P) in Canada.

Our study was designed on the basis of a potentially large sample of adolescent girls, given that FitSpirit works with more than 250 Canadian schools to implement PA interventions for nearly 12,000 girls annually. For the research component, ten participants from each participating school were randomly selected through a draw. Pre-participation assessments were conducted between December 2018 and March 2019, and post-participation assessments were completed in May and June 2019. After the registration period and at the end of the school year, FitSpirit sent an online survey by email to the selected girls.

### 2.2. Assessments and Measures

A 35 question, self-reported questionnaire, available online in both French and English, was used pre- and post-participation. The questionnaire required approximately 30 min to complete, and participants recorded their responses online during their time outside school.

The demographic questions assessed participants’ ages, weight and height. Age- and sex-specific zBMI values were calculated according to the World Health Organization guidelines [44]. Questions assessing the adolescents’ physical activity levels and lifestyle habits were adapted from two validated self-reported surveys [45,46]. The questionnaire collected information on the frequency and duration of PA during a typical week and covered both active transportation and leisure time PA. PA level was assessed by asking how many days per week each participant performed a total of at least 60 min of MVPA (none to every day) as used elsewhere [47]. For evaluating sedentary behaviors, participants were asked to provide their average number of daily hours and minutes spent watching television, playing video and computer games, and browsing the Internet. The questionnaire assessed participants’ sleeping habits by asking the average number of daily hours of sleep as used elsewhere [48]. The adherence to recommendations for PA levels, screen time and sleep was assessed by using the daily times recommended according to age in the Canadian 24-Hour Movement Guidelines for Children and Youth [1]. The dietary questions included how many days per week (0–7), and how many servings of the following ten food groups were consumed: vegetables, fruits, fruit and vegetable juices, milk, cheese, yogurt, water, sugar-sweetened beverages, sweets and fast food. For evaluation of breakfast intake, participants were asked how many weekdays they ate and/or drank something before going to school in the prior school week as used elsewhere [49]. The adherence to dietary guidelines for consumption of vegetables and fruits was assessed on the basis of the 2007 Canada’s Food Guide recommendations for age and sex [22]. All questions and scales are presented in Appendix A.

### 2.3. Data Analysis

Descriptive statistics were used for demographic characteristics and pre- and post-participation values for PA levels, sedentary behaviors, and sleeping and eating habits. Independent sample t tests were used to compare the demographic characteristics of participants according to the number of FitSpirit activities completed in the pre-participation survey. Only data from girls who participated in five or fewer FitSpirit activities before enrollment were included in the analyses for primary and secondary outcomes.

Considering that the data were normally distributed, paired sample t-tests were used to compare mean differences in pre- to post-participation values for lifestyle habits (including PA, sedentary behaviors, and sleeping and eating habits). Fisher’s exact test was conducted to evaluate the differences in PA level changes between groups, after stratification by activity level pre-participation (inactive: cumulative 60 min of MVPA < 4 days/week and active: cumulative 60 min of MVPA at least 4 days/week). Independent sample t tests were then used to evaluate the differences in PA, screen time, sleep duration and eating habit changes between groups, after stratification by adherence (or lack thereof) to their corresponding Canadian recommendations before enrollment. Cohen’s *d* was used to report the effect size. SPSS 25.0 (SPSS Inc., Chicago, IL, USA) was used for analyses.

## 3. Results

**Figure 1:**
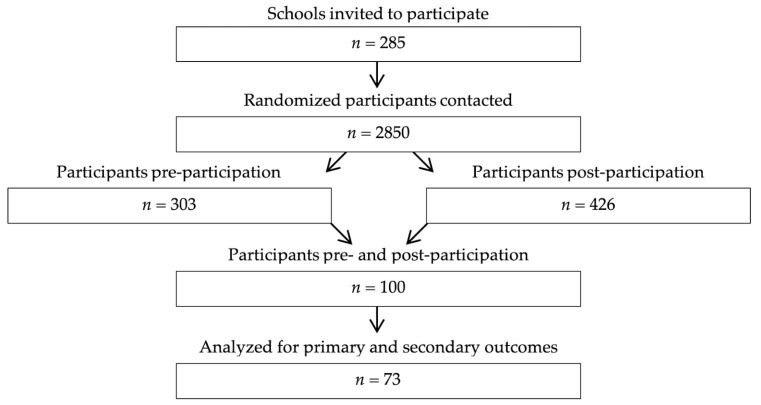
Flowchart of participants through the study.

Of the 2850 girls invited to participate, 3.5% completed both the pre- and post-participation questionnaires (Figure 1). Participants were on average aged 14.9 ± 1.5 years old, were 1.61 ± 0.10 m in height, weighed 57.2 ± 13.8 kg and had a zBMI of 0.41 ± 0.92. The number of active days was 30% lower in girls who had taken part in five FitSpirit activities or fewer than in girls who had taken part in more than five activities before enrollment (Table 2). Thus, only data from girls who participated in five or fewer FitSpirit activities before enrollment were included in the analyses.

### 3.1. Primary Outcomes

There was a significant increase (19%) in the number of active days, with a small effect size. However, the time spent on active commuting and leisure-time PA did not change (Table 3).

### 3.2. Secondary Outcomes

There was a 34% decrease in the consumption of sweets, with a small effect size (Table 4). No other significant changes in lifestyle habits were observed.

### 3.3. Subgroup Analysis

Subgroup analysis based on the adherence to recommendations for lifestyle habits revealed subgroup differences. Girls who were less active before enrollment increased their number of active days, whereas those who were active before enrollment decreased their number of active days, with a large effect size (Table 5).

**Figure 2:**
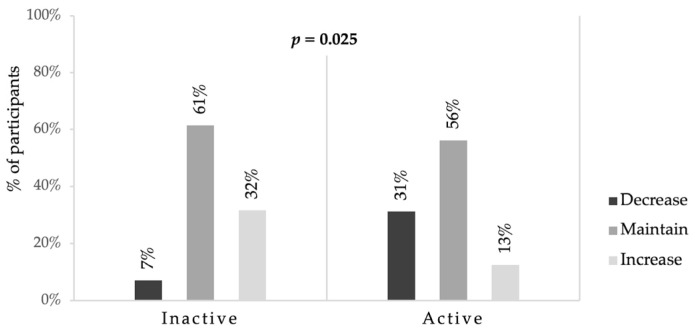
Changes in physical activity levels by activity level before enrollment. Note: *p: p*-value; inactive: reach PA recommendations < 4 days/week; active: reach PA recommendations at least 4 days/week; PA: physical activity [1].

Inactive girls before enrollment were more likely to increase their PA levels than girls who were already active most days (Figure 2).

Girls who did not meet the recommendations for the behaviors evaluated before enrollment were more likely to improve their lifestyle behaviors, including decreasing screen time, with a moderate effect size, and increasing fruit and vegetable consumption, with large effect sizes (Table 6 and Table 7). Change in yogurt consumption was higher in the non-adherent group than the adherent group, with a moderate effect size.

## 4. Discussion

Several school-based PA interventions have aimed to increase PA among adolescents. Most studies have evaluated the interventions’ effects on PA levels, but very few have also assessed lifestyle changes, such as eating habits and sleep patterns [40,41]. To our knowledge, this is the first study to evaluate the effects of a PA intervention on a wide range of lifestyle habits, including PA, screen time, sleep patterns and eating habits, in a Canadian multi-component program designed to help schools implement PA intervention aimed at adolescent girls.

Overall, FitSpirit participants increased their number of active days per week and decreased their consumption of sweets relative to baseline. There were no changes in other health behaviors. Subgroup analysis revealed that girls who did not have a cumulative 60 min of MVPA at least 4 days per week before enrollment had the greatest improvements in PA relative to those who were more active. This result was reflected by an increase in the number of active days, which was 1.2 days higher in the inactive group than the active group. This change would not have been significant if it had been subjected to a multiplicity test. However, because the effect size was large, and inactive girls are the target population for FitSpirit, it is relevant to highlight this result. Only participants who had screen time of at least 2 h daily at baseline reduced their screen time, and this change was 1.25 h less than that of participants who already met the screen time recommendation at baseline. Because PA levels decrease while sedentary behaviors increase with age in Canadian girls, the finding that girls were active more frequently after FitSpirit participation is relevant [50]. The intervention had no impact on sleep duration, although sleep has previously been associated with physical activity [48]. This finding is probably due to the fact that FitSpirit mainly aims to increase physical activity in adolescent girls and does not address the theme of sleep. Participants who did not meet dietary guidelines at baseline increased their fruit and vegetable intake over the course of the program, by 0.3 and 0.7 servings daily, respectively, and maintained their yogurt consumption, which was 0.4 daily servings higher than that of participants adhering to dietary guidelines before enrollment. Adolescent girls in the Canadian population tend to consume increasingly fewer servings of vegetables and fruits and dairy products, therefore, the increase and maintenance of consumption of these food groups, respectively, after FitSpirit participation is an encouraging change [23].

Associations among lifestyle habits have previously been studied in adolescents. Torstveit et al. showed that participating in organized sports is inversely associated with sedentary behaviors, short sleep duration and unhealthful eating habits, such as irregular consumption of main meals and high intake of unhealthful food and beverages [48]. Active adolescents are more likely to engage in healthful dietary behaviors then their inactive peers. Duncan et al. also suggested that activity status (active vs. inactive) appears to play an important role in other lifestyle habits [51]. In the current study, the interventions mainly focused on PA-related behaviors, and positively affected different lifestyle habits. Our findings support those of previous reports that show that changes in PA levels often occur along with changes in screen time, and daily intake of sweets, fruits and vegetables. Thus, when an intervention is aimed at one health-related behavior, it is important to measure others lifestyle habits. However, this study also shows that it may be difficult to have an impact on a wide range of lifestyle habits including sleep duration and other eating habits such as eating breakfast. Future interventions that target physical activity should include components focused on these lifestyle habits or be offered in schools in complementary interventions that target other lifestyle habits.

The results of this study are also consistent with those of other studies on multi-component programs focusing on lifestyle changes in children and adolescents. In a multi-component 20-month intervention targeting participants’ overall PA and sedentary behavior, Grydeland et al. reported an increase in PA in adolescents in the low-activity group (i.e., 92 counts per min) as compared with that in the high-activity group [52]. These results corroborate those from the current study, in which changes in PA levels were greater in the inactive group. This result can be explained by several contextual factors, including a greater potential for an increase in PA. In a six-week program providing 45 min of structured PA and a 45 min nutrition education class for children, both boys and girls increased their participation in PA to at least 60 min/day, and the effects were sustained during a 12-month follow-up (i.e., 2.52 and 2.49 days/week) [47]. Those changes in PA levels are greater than observed in our study (i.e., 0.5 day/week), thus highlighting that increasing PA levels appears to be more difficult in adolescent girls, as shown by intervention studies targeting girls in school settings.

GoGirlGo! is a 12-week program, with 1-h lessons including 30 min of education on specific developmentally appropriate life skill topics and 30 min of PA weekly. Among participants, on days when the interventions were delivered, a greater cumulative MVPA during the time of program attendance was recorded on data collection days than on days without activities (i.e., 2.5 and 2.9 min/day, respectively) [53]. Participants with a greater proportion of time in MVPA (+5%) were more inclined to spend a lower proportion of time on sedentary activities (5–7 years, 58% vs. 53%; 8–10 years, 62% vs. 55%, for children with lower and higher MVPA, respectively). In this study, the changes were not sustained after the 12-week program. Some studies targeting girls have not observed a significant effect on PA but have shown a decrease in sedentary behaviors [40,54,55]. The intervention proposed by FitSpirit has multiple components and considers barriers and facilitators of PA in adolescent girls, such as including girls-only activities that are diverse, non-competitive, fun and performed with friends [56]. Moreover, Laroche et al. have shown promising results regarding how FitSpirit can improve participants’ motivation for subsequent PA [43]. Thus, our results corroborate these studies’ findings and show that FitSpirit can contribute to limiting the decrease in PA and the increase in sedentary behavior among Canadian girls, especially among less active girls and those who spend the most time using a screen.

Our study is the first to evaluate the effects of the interventions from this program, which was implemented more than 10 years ago and promotes PA among adolescent girls. Integrating the current study’s evaluation methodology without interfering with the program’s daily operations was challenging. Consequently, this research is subject to some limitations. First, the experimental design did not allow for a control group, because all study participants were registered for FitSpirit. Second, adolescent girls were asked to respond to online questionnaires, and the invitation occurred after school registration with FitSpirit. Schools were allowed to register over the course of 5 months, and some schools had already started FitSpirit activities when the participants answered the initial questionnaire. Third, the reliance on self-reported lifestyle habits may have increased the risk of over- or underestimation, desirability bias and recall bias, and girls who responded may have had greater motivation to participate in the program. Given the scope of FitSpirit and the logistical constraints, the use of questionnaires was the method best suited for its ease of use and for the facilitated outreach. Fourth, a seasonal bias should be considered, because the end of the intervention coincided with the end of the school year. A trend toward a decrease in sleep time and in the number of active days among participants meeting recommendations at baseline could be explained by the exam period that occurred between May and June. Changes in weather conditions between winter and summer, especially in eastern Canada, may also explain changes in lifestyle habits, such as an overall increase in active days per week. Finally, lifestyle habits including sleep duration and time could be associated with numerous factors that were not measured in the study.

## 5. Conclusions

Our findings suggested that participation in FitSpirit potentially contributes to improving certain health behaviors among adolescent girls, particularly among those who did not adhere to the Canadian recommendations at baseline. Overall, participants reported improvements following the intervention, with an increase in the number of active days and a decrease in the daily consumption of sweets. Participants who were less active before enrollment showed greater increases in their number of active days. Those who spent the most time using a screen before enrollment decreased their screen time. Participants who did not meet an important dietary guideline (vegetable and fruit consumption) before enrollment increased their intakes and maintained their yogurt consumption, whereas participants adhering to those guidelines decreased their yogurt consumption. The decrease in the consumption of sweets is interesting, because these results have rarely been reported in the context of a school-based PA intervention. There was no change in sleep duration and others eating habits. Studies with longer follow-up are needed to understand whether and how these changes can be sustained over time. Future interventions promoting healthful behaviors among adolescent girls should target change in multiple lifestyle factors and assess health-related quality of life including mental health and wellness among participants.

## Figures and Tables

**Table 1:** Components of the FitSpirit program.

Component	Description
Motivational conferences	At the beginning of the intervention, schools can receive visits from FitSpirit ambassadors to increase girls’ motivation to pursue an active lifestyle and to register in the program.
Running program	A turnkey running training program is available to help schools provide planned PA sessions to prepare girls for a running event at the end of the school year.
Special PA sessions	School leaders can choose up to three FitSpirit ambassadors every year to lead PA sessions in which various activities and sports can be offered.
Special events	At the end of the school year, FitSpirit organizes celebration days in several regions, during which a variety of activities and sports are offered, and girls participate in either a self-paced 5 K or 10 K run.
Webinars and online tools	FitSpirit offers online tools including an interactive tool to help girls create their own workouts, access tips, and connect with a dietitian and a kinesiologist to answer their nutrition and PA questions. School leaders and ambassadors also have access to a web platform containing more tools.

Note: PA, physical activity.

**Table 2:** Characteristics of participants according to the number of FitSpirit activities completed before enrollment.

Characteristic	≤5 Activities		>5 Activities		*p*	*d*
	*n*		*n*			
Age (years)	73	14.7 ± 1.6	23	15.4 ± 1.3	0.056	0.462
Weight (kg)	68	55.9 ± 12.2	19	58.5 ± 12.8	0.421	0.210
Height (m)	70	1.60 ± 0.10	22	1.63 ± 0.07	0.362	0.224
BMI Z-score	66	0.37 ± 0.92	18	0.42 ± 0.79	0.825	0.059
Active days (days/week)	73	2.7 ± 1.5	23	3.5 ± 1.6	**0.030**	0.528

Note: Data are presented as mean ± standard deviation. *p*: *p*-value; BMI: body mass index; *d*: Cohen’s *d*. Result in bold is statistically significant (*p* < 0.05).

**Table 3:** Physical activity pre- and post-participation.

Physical Activity Outcome	*n*	Pre	Post	*p*	*d*
Active days (days/week)	73	2.7 ± 1.5	3.2 ± 1.6	**0.024**	0.270
Active commuting (min/week)	67	122 ± 136	138 ± 117	0.308	0.126
Leisure-time PA (min/week)	71	219 ± 174	217 ± 164	0.908	0.014

Note: Data are presented as mean ± standard deviation. *p*: *p*-value; *d*: Cohen’s *d*. Result in bold is statistically significant (*p* < 0.05).

**Table 4:** Screen time, sleep duration and eating habits pre- and post-participation.

Lifestyle Habit	*n*	Pre	Post	*p*	*d*
Screen time (h/day)	72	3.45 ± 2.12	3.44 ± 1.82	0.950	0.007
Sleep duration (h/day)	72	8.47 ± 1.29	8.42 ± 1.51	0.761	0.036
Eating habits (servings/day)					
Fruits	72	1.8 ± 1.1	1.6 ± 1.0	0.070	0.216
Vegetables	72	1.7 ± 1.0	1.6 ± 1.0	0.821	0.027
Fruit/vegetable juices	72	0.9 ± 0.8	0.8 ± 0.9	0.232	0.142
Milk	70	1.2 ± 1.2	1.1 ± 1.1	0.178	0.163
Cheese	71	1.0 ± 1.0	0.9 ± 0.9	0.144	0.175
Yogurt	71	0.6 ± 0.8	0.5 ± 0.7	0.076	0.214
Water	65	3.1 ± 1.2	2.9 ± 1.2	0.067	0.231
Sugar-sweetened beverages	72	0.3 ± 0.5	0.3 ± 0.5	0.936	0.010
Sweets	70	0.8 ± 0.9	0.5 ± 0.5	**0.006**	0.341
Fast food	71	0.2 ± 0.3	0.2 ± 0.2	0.741	0.023
Breakfast (days/weekdays)	72	3.8 ± 2.0	4.0 ± 1.9	0.113	0.097

Note: Data are presented as mean ± standard deviation. *p*: *p*-value; *d*: Cohen’s *d*. Result in bold is statistically significant (*p* < 0.05).

**Table 5:** Changes in physical activity outcomes by pre-participation activity level.

Change in Physical Activity Outcome	*n*	Inactive	*n*	Active	*p*	*d*
△ Active days (days/week)	57	0.7 ± 1.4	16	−0.5 ± 1.5	**0.004**	0.833
△ Active commuting (min/week)	53	29 ± 124	14	−30 ± 146	0.132	0.458
△ Leisure-time PA (min/week)	57	5 ± 166	14	−32 ± 250	0.510	0.197

Note: Data are presented as mean ± standard deviation. *p*: *p*-value; *d*: Cohen’s *d*; Inactive: reach PA recommendations < 4 days/week; active: reach PA recommendations at least 4 days/week; △: changes pre- to post-participation; PA: physical activity [1]. Result in bold is statistically significant (*p* < 0.05).

**Table 6:** Changes in screen time and sleep duration according to participants’ adherence to recommendations pre-participation.

Change in Screen and Sleep Time	*n*	Non-Adhering	*n*	Adhering	*p*	*d*
△ Screen time (h/day)	48	−0.46 ± 1.92	23	0.79 ± 1.44	**0.007**	0.533
△ Sleep duration (h/day)	26	0.02 ± 1.96	46	−0.09 ± 0.87	0.752	0.078

Note: Data are presented as mean ± standard deviation. *p*: *p*-value; *d*: Cohen’s *d*; non-adhering: below associated recommendations; adhering: reach associated recommendations; △: changes pre- to post-participation [1]. Result in bold is statistically significant (*p* < 0.05).

**Table 7:** Changes in eating habits according to participants’ adherence to dietary guidelines pre-participation.

Change in Eating Habit	*n*	Non-Adhering	*n*	Adhering	*p*	*d*
△ Eating habits (servings/day)						
Fruits	62	0.3 ± 2.4	10	0.0 ± 2.2	**0.001**	1.202
Vegetables	62	0.1 ± 0.7	10	−0.6 ± 0.8	**0.003**	1.032
Fruit/vegetable juices	62	0.0 ± 0.7	10	−0.5 ± 1.0	0.054	0.669
Milk	61	−0.1 ± 1.1	9	−0.8 ± 1.2	0.057	0.692
Cheese	62	−0.1 ± 0.7	9	−0.6 ± 1.1	0.104	0.877
Yogurt	62	−0.1 ± 0.4	9	−0.5 ± 1.0	**0.016**	0.588
Water	55	−0.2 ± 1.0	10	−0.4 ± 0.5	0.468	0.251
Sugar-sweetened beverages	62	0.0 ± 0.4	10	0.2 ± 0.3	0.156	0.489
Sweets	61	−0.2 ± 0.8	9	−0.3 ± 0.7	0.739	0.119
Fast food	62	0.0 ± 0.2	9	0.0 ± 0.1	0.619	0.178
△ Breakfast (days/weekdays)	62	0.3 ± 2.2	10	0.0 ± 2.4	0.743	0.112

Note: Data are presented as mean ± standard deviation. *p*: *p*-value; *d*: Cohen’s *d*; non-adhering: below dietary guidelines for vegetables and fruits; adhering: reach dietary guidelines for vegetables and fruits; △: changes pre- to post-participation [49]. Results in bold are statistically significant (*p* < 0.05).

## Appendix A. Questions and Scales Used Pre- and Post-Participation

- What are the year and month of your birth?
- Over a typical or usual week, on how many days are you physically active for a total of at least 60 min in total per day? Consider only the activities that made you breathe harder and sweat at least a little. [46]
  ○ None (zero days)
  ○ 1 day
  ○ 2 or 3 days
  ○ 4 to 6 days
  ○ Everyday
- In the last 7 days, how long did you use active forms of transportation to get around, like walking to school or cycling to get to work, the shopping center or a friend’s place? (Enter hours AND minutes. You can enter 0 h and 0 min if you have not used active modes of transportation.) [46]
- In the last 7 days, how long did you do physical activity in your leisure time including exercising, playing an organized or non-organized sport or playing with your friends? (Enter hours AND minutes. You can enter 0 h and 0 min if you have not been active during your leisure time.) [46]
- On average, about how many hours a day do you spend in front of a screen? Include time spent on a computer, watching TV or videos or playing video games. (Enter hours AND minutes.) [46]
- How many days a week do you eat from each food group [45]
  	**Never**	**1 d/week**	**2 d/week**	**3 d/week**	**4 d/week**	**5 d/week**	**6 d/week**	**7 d/week**
  Whole fruit	1	2	3	4	5	6	7	8
  Whole vegetable	1	2	3	4	5	6	7	8
  Fruit and vegetable juices	1	2	3	4	5	6	7	8
  Milk (or fortified soy beverage)	1	2	3	4	5	6	7	8
  Cheese (all kinds)	1	2	3	4	5	6	7	8
  Yogurt (all kinds)	1	2	3	4	5	6	7	8
- On the days you eat or drink of each group, how many servings do you usually have? [45]
  	**None**	**1 Serving**	**2 Servings**	**3 Servings**	**4 Servings or more**
  Whole fruit (1 serving = a fruit the size of a tennis ball or medium size)	1	2	3	4	5
  Whole vegetable (1 serving = a medium size; 1 cup of leafy vegetables or salad)	1	2	3	4	5
  Fruit and vegetable juices (no added sugar; 1 serving = 125 mL or ½ can of juice)	1	2	3	4	5
  Milk (or enriched soy beverage; 1 serving = 1 cup)	1	2	3	4	5
  Cheese (all kinds; 1 serving = 50 g or about the size of two fingers of cheddar, feta cheese; 1 cup of cottage cheese)	1	2	3	4	5
  Yogurt (all kinds; 1 serving = ¾ cup yogurt, yogurt to drink or kefir)	1	2	3	4	5
- How many days a week do you eat from each food category ? [45]
  	**Never**	**1 d/week**	**2 d/week**	**3 d/week**	**4 d/week**	**5 d/week**	**6 d/week**	**7 d/week**
  Glasses of water	1	2	3	4	5	6	7	8
  Sugary drinks (fruit cocktails, sport drinks, energy drinks, etc.)	1	2	3	4	5	6	7	8
  Sweets (candy, chocolate, popsicles, etc.)	1	2	3	4	5	6	7	8
  Fast Food (McDonald’s, Burger King, etc.)	1	2	3	4	5	6	7	8
- On the days you eat or drink of each category, how many servings do you usually have? [45]
  	**None**	**1 Serving**	**2 Servings**	**3 Servings**	**4 Servings or more**
  Glasses of water (1 serving = 1 cup or 250 mL)	1	2	3	4	5
  Sugary drinks (1 serving = a can; 1 cup or 250 mL of Coke, fruit punch, Gatorade, Re Bull, etc.)	1	2	3	4	5
  Sweets (1 serving = 1 popsicles; a handful of candy, chocolate, etc.)	1	2	3	4	5
  Fast Food (1 serving = meal at McDonald’s, Burger King, etc.)	1	2	3	4	5
- During the past school week (Monday to Friday), how many days did you eat or drink something in the morning (including breakfast) before school began? Don’t count coffee, tea or water. [45]
- How many hours do you usually spend sleeping in a 24-h period? (Enter hours AND minutes.) [46]
- How tall are you without shoes on? You can choose to answer in meters and centimeters OR in feet and inches. [46]
- How much do you weigh? You can choose to answer in kilograms OR pounds. [46]
- How many FitSpirit activities have you participated in since the start of the school year? (adds training, ambassador visits, outings, conferences, etc.)
  ○ None
  ○ 1 to 5
  ○ 6 to 10
  ○ 11 to 15
  ○ 16 to 20
  ○ 21 to 25
  ○ 26 or more
